# Representation of vocalizations in the frontal auditory field and the dorsal auditory cortex of bats

**DOI:** 10.1111/nyas.15336

**Published:** 2025-04-08

**Authors:** Stephen Gareth Hoerpel, Sonja C. Vernes, Uwe Firzlaff

**Affiliations:** ^1^ TUM School of Life Sciences Technical University of Munich Freising Germany; ^2^ School of Biology University of St Andrews St Andrews UK

**Keywords:** auditory cortex, auditory neuroscience, electrophysiology, frontal cortex, vocal communication

## Abstract

In bats, which express a complex vocal repertoire and are considered vocal learners, the frontal auditory field (FAF) is supposedly placed in a frontal cortico‐striatal network for vocal–motor control. The FAF receives input from the auditory cortex (AC) and other auditory nuclei via multiple pathways. However, not much is known about the transition of information on vocalizations from the AC to the FAF. The bat AC consists of different subfields, among which the dorsal fields (dAC) are characterized by precise coding of the temporal envelope of vocalizations. The dAC should, therefore, be a major source of auditory feedback information about self‐produced or perceived vocalizations to the FAF. Our study aimed to investigate the specificity of encoding for different types of vocalizations in FAF and dAC neurons. Using extracellular recordings in anesthetized *Phyllostomus discolor*, we describe basic response properties in both cortical areas and compare responses to different types of prerecorded vocalizations. The specificity of encoding for different behaviorally relevant call categories and single calls was higher in dAC than in FAF neurons, both in terms of temporal firing patterns and response strength. These findings highlight the importance of the dAC in the neural network for control of vocal communication in bats.

## Graphical abstract

This study investigated the specificity of encoding for different types of vocalizations in neurons of the frontal auditory field (FAF) and the dorsal auditory cortex (dAC) of a bat. Our results show that dAC neurons were more selective for call categories and single calls than neurons in the FAF. This contrasts with earlier findings and highlights the importance of the dAC in the neural network for control of vocal communication in bats.

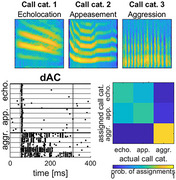

## INTRODUCTION

Bats express a rich vocal repertoire for social communication and are among the few mammalian species capable of vocal learning.^1^ ^−^ ^4^ Extensive work on the ascending auditory pathway has been undertaken in bats, mostly investigating bio‐sonar‐related functions (e.g., Ref. 5). By contrast, considerably less research has investigated frontal cortical areas that might be involved in control of vocalizations or serving higher auditory functions in bats. However, these are particularly relevant as the prefrontal cortex is considered an important region in the network enabling volitional control of vocalizations in mammals (both primate and nonprimate)^6^ and consequently is also considered an important substrate for vocal learning in mammals.^7^ In bats, most work on frontal cortical areas responsive to auditory stimulation have focused on the frontal auditory field (FAF) of *Pteronotus parnellii*,^8^ ^−^ ^10^ *Carollia perpicillata*,^11^ ^−^ ^14^ *Tardarida brasiliensis*,^15^ *Roustettus aegyptiacus*,^16^ and *Phyllostomus discolor*.^17^ Neuroanatomical tracer studies in *P. parnellii* demonstrated direct projections from a division of the auditory thalamus, the supra‐geniculate nucleus, to the FAF,^8^ therefore, labeling the FAF as a target region of the extra‐lemniscal pathway.^9^ In *P. discolor*, localization of the FAF and parts of its connectivity to cortical and subcortical areas have been described.^17^ In general, these data show that the FAF may broadly serve to link auditory and motor systems as it projects to the superior colliculus,^8^ a major hub for sensory‐motor integration^18^ but also to the pyramidal tract, a known motor‐control pathway.^17^ However, the FAF also receives direct input from the auditory cortex (AC).^8^, ^17^, ^19^ As the AC is the highest stage of the ascending auditory pathway and a major hub for auditory processing, it should also play an important role in the cortico‐striatal network for audio‐vocal–motor integration. Interaction between frontal and auditory cortices may contribute to mechanisms that compare intended and actual vocal outputs during vocal communication and learning.^20^ Furthermore, García–Rosales et al.^13^ demonstrated coherent sound‐onset evoked gamma oscillations in the FAF and the AC, suggesting a functional coupling between these two auditory brain regions.

So far, not much is known about the transition of information on vocalizations from the AC to the FAF. Therefore, we aimed to compare the basic response properties of neurons in the FAF and the dorsal auditory cortex (dAC; defined as the anterior‐dorsal field and the posterior‐dorsal field of the AC)^21^ as well as responses to different types of prerecorded vocalizations (echolocation calls and two types of communication calls) in anesthetized *P. discolor*. We focused on the dAC as neurons in this region often show precise spike timing and can encode fast amplitude modulation patterns of communication calls.^22^ Furthermore, dorsal cortical areas in bats often show precise spike timing, as they are partly involved in processing echo‐delay information important for target distance detection during echolocation. The precise measurement of the delay between call and returning echo might also favor the processing of combinations of different syllables in vocal communication.^23^, ^24^ In addition, areas located dorsally to the primary auditory cortex (A1) in the mouse are sensitive to communication sounds,^25^ especially in the behavioral context of pup retrieval.^26^

In particular, we investigated if neurons in both areas could specifically encode calls from a particular call category. This is of special interest, as García–Rosales et al.^27^ showed that the dynamics of information flow between the FAF and the AC predicted the purpose of a vocalization (communication or echolocation) in vocalizing bats, indicating call category‐specific neuronal mechanisms. While Macias et al.^15^ found the FAF in *T. brasiliensis* to be more selective for communication calls than the A1, our results in *P. discolor* suggest the opposite—responses in the FAF were less specific for call categories or single calls than neurons in the dAC.

## METHODS

### Surgery

All experiments complied with the principles of laboratory animal care and were conducted following the regulations of the current version of the German Law on Animal Protection (approval ROB‐55.2‐2532.Vet_02‐13‐147, Regierung von Oberbayern). The bats (*P. discolor*; three adult females, one adult male were used for recordings in the FAF, three adult females and one adult male bat for the dAC recordings) originated from a breeding colony situated in the Department Biology II of the Ludwig‐Maximilian University of Munich. For experiments, animals were kept separated from other bats under semi‐natural conditions (12 h day/12 h night cycle, 65%–70% relative humidity, 28°C) with free access to food and water.

The surgical procedures are described in detail in previous publications,^21^, ^22^ and details of the stereotaxic device and the procedure used to reconstruct the recording sites are described.^28^ Briefly, the bats were anesthetized using a combination of medetomidine (Dorbene; Zoetis), midazolam, (Dormicum; Hoffmann‐La Roche), and fentanyl (Fentadon; Albrecht) at dosages of 0.4, 4.0, and 0.04 µg/g body weight, respectively. Anesthesia was maintained through additional injections containing two‐thirds of the initial dose every 1.5 h. The skin overlying the skull was opened along the midline, and the skull surface was freed from tissue. A small metal tube was then fixed to the skull using a microglass composite to fix the animal to a stereotaxic device. The alignment of the animal's skull and the underlying brain within the stereotaxic coordinate system was measured by scanning the characteristic profile lines of the skull in the parasagittal and frontal planes. These profiles were then digitally fitted to a standardized skull profile in a standardized coordinate system.

To alleviate postoperative pain, an analgesic (0.2 µg/g body weight; meloxicam, Metacam, Boehringer‐Ingelheim) was administered after the surgery for 4 postoperative days. The anesthesia was antagonized with a mixture of atipamezole (Alzane, Novartis), flumazenil (flumazenil, Hexal), and naloxone (Naloxon‐ratiopharm, Ratiopharm), which was injected subcutaneously (2.5, 0.5, and 1.2 µg/g body weight, respectively). The bats were treated with antibiotics (0.5 µg/g body weight; enrofloxacin, Baytril, Bayer AG) for 4 postoperative days.

### Acoustic stimulation

Acoustic stimuli were presented using either Brain Ware (Tucker‐Davis Technologies, TDT) for recordings in the dAC or AudioSpike (HörTech gGmbH) for recordings in the FAF.

All acoustic stimuli were computer‐generated (MATLAB; The MathWorks), digital–analog converted (either RX6 [TDT] or Fireface 400, RME; sampling rate 195.312 Hz with the RX6 or 192 kHz with the FireFace 400), amplified (AX‐396, Yamaha Music Foundation) and presented via free‐field loudspeaker (R2904/700000, Scan‐Speak). The loudspeaker had been calibrated for linear frequency response between 1 and 96 kHz using a ¼ inch ultrasonic reference microphone (Type 4939 microphone attached to a Type 2610 measuring amplifier, Brüel & Kjær). The loudspeaker was positioned contralaterally ∼30° off the head midline at a distance of ∼20 cm. Stimuli to search for neuronal activity (typically social communication calls or noise signals) were presented with a repetition rate of 2 Hz.

A frequency response curve of a neuron under study was established by presenting pure tone stimuli of 20 ms duration, with a frequency range from 5 to 80 kHz (logarithmically spaced in 1/8 octave steps) at sound pressure levels (SPL) from 80 to 15 dB re 20 µPa. The pure tones were presented with a 2 Hz repetition rate in random order and repeated 10 times. Each stimulus presentation was preceded by a 50 ms silent interval.

Additionally, each neuron was presented with *P. discolor* echolocation and communication calls with 20 repetitions per call in random order at a level of 15−20 dB above the neuronal response threshold. The calls were selected out of a database recorded in the *P. discolor* colony at the Department of Biology II of the Ludwig Maximilians University Munich. The selected calls were acoustically representative of the most common call types associated with social behaviors (aggressive and nonaggressive social interactions) and echolocation (see below).

**FIGURE 1:**
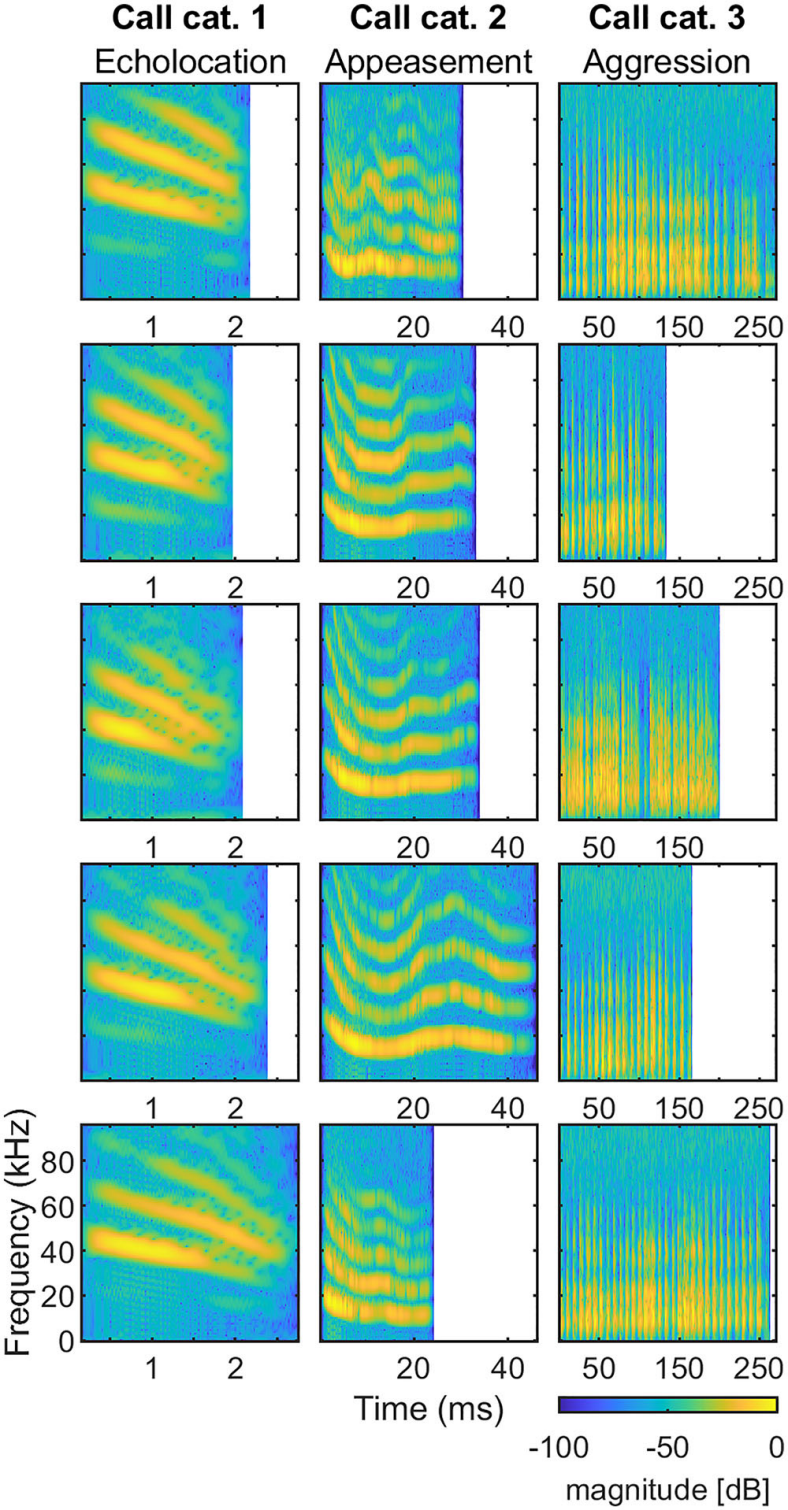
Spectrograms of the 15 prerecorded *Phyllostomus discolor* vocalizations used for acoustic stimulation. Vocalizations are grouped into three categories (echolocation, appeasement, and aggression) based on the behavioral context during which they are typical emitted by the bats (see Lattenkamp et al., 2019 for details). Note the different time scales for the different call categories. Abbreviation: cat., category.

A total of 15 calls (Figure 1) was grouped into three categories, roughly following the classification of Lattenkamp et al.^29^ and on the basis of differences in several acoustic parameters such as duration, fundamental frequency *F*0, roughness, and occurrence of amplitude and frequency modulations in the call envelope and spectrogram, respectively.

Category 1 (Figure 1, first column) consisted of typical echolocation calls, therefore, short in duration (<3 ms) and multiharmonic downward frequency modulated in the range between approximately 90 and 40 kHz.

Category 2 (Figure 1, second column) calls had a duration between 25 and 46 ms, had a strongly harmonic spectrum with a fundamental frequency of about 17 kHz, and displayed shallow frequency modulations. The calls were very similar to mother–pup contact or appeasement calls, as shown by Esser and Schmidt,^30^ and also resembled those labeled as “SFM” in Lattenkamp et al.^29^ that were reported to be used in nonaggressive social interactions.

Category 3 (Figure 1, third column) consisted of long (134−271 ms) calls with a varying number of short broadband sound elements separated by approximately 8 ms and thus also giving rise to strong amplitude modulations. In contrast to category 2, category 3 calls had a lower fundamental frequency of approximately 7.5–13 kHz. These calls are usually associated with aggressive behavior toward conspecifics.^29^

We analyzed and compared four acoustic call features: spectral centroid, temporal centroid (i.e., the centroid of the temporal envelope), duration, and harmonic ratio, which indicates the ratio of energy in the harmonic portion of the call to the total energy of the call. Figure S1 shows that for each of these acoustic features, at least one call category forms a separate cluster. Thus, the call categories are well distinguishable based on these parameters. There is also some degree of inner‐category variability of acoustic call features.

Earlier studies on neural coding of communication calls made use of pitch‐shifted variants of calls (e.g., Ref. 31) to cover the interindividual variation of fundamental frequencies of calls. As this might be more important for constant frequency bats such as *P. parnellii* than for frequency‐modulated (FM) bats such as *P. discolor*, we have refrained from using this method here.

### Electrophysiological recordings

After initial surgery, experiments were conducted in a sound‐attenuated and heated (∼35°C) chamber. Extracellular recordings were made with parylene‐coated tungsten microelectrodes (5MOhm impedance; Alpha Omega) in anesthetized bats (see Surgery). Note that the responses recorded from cortical units under this anesthesia regime reflect the behavioral performance of *P. discolor* well.^32^ Recording sessions took place 3 days per week for up to 8 weeks (with at least 1 day off between consecutive experiments) and could last up to 5 h per day. Dorso‐ventral electrode penetrations in the dAC and the FAF were run obliquely to the brain surface with different mediolateral and rostrocaudal angles. The electrode signal was recorded using an analog‐to‐digital converter (TDT RA16) and either a combination of RX5 and Brain Ware (sampling rate 25 kHz, bandpass filter 400–3000 Hz, TDT) or a FireFace400 and Audiospike (sampling rate 24 kHz, bandpass filter 400–3000 Hz). The action potentials were threshold discriminated and saved for later offline analysis. We tried to isolate single neurons whenever possible; however, it was not always possible to clearly discriminate the activity of a single neuron. Therefore, the term “unit” is used in this article to describe the collective activity of one to three neurons recorded at a recording site.

After the experiments were completed, a neuronal marker (BDA 3000; Sigma‐Aldrich; 1 mg/20 µL phosphate buffer) was pressure‐injected (Nanoliter 2010 injector; World Precision Instruments) into the brains to reconstruct the position of the recording sites in standardized stereotaxic coordinates^28^ of a brain atlas of *P. discolor*.^33^ Finally, the animals were euthanized by an intraperitoneally applied lethal dose of pentobarbital sodium (0.16 mg/g body weight) and transcardially perfused with 4% paraformaldehyde for later histological processing. Brains were stored at −80°C. Forty micrometer slices were done with an HM 440E microtom (Microm GmbH). Slices were mounted on slides covered with chromalaun gelatine (Sigma‐Aldrich) and dried overnight. Nissl stainings were done with 1.5% cresyl‐violet (pH 3.9, Roth). In detail, slices were rinsed three times for 1 min with distilled water and stained with 1.5% cresyl‐violet for 15 s. Slices were dehydrated for 30 s in 95% ethanol (CLN GmbH) and two times for 30 s in 100% ethanol (CLN GmbH). The alcohol was removed by rinsing two times for 5 min in xylol (CLN GmbH). Slices were cover slipped with DPX (Sigma‐Aldrich).

### Data analysis

#### Responses to pure tones

All analyses were done with MATLAB. Initially, we calculated both the best frequency (BF, frequency with the strongest neuronal response) and characteristic frequency (CF, frequency at which a given unit responds to the lowest sound intensity) with their respective SPL values. This was achieved by calculating the median neuronal response rate (over 10 repetitions) for every frequency and SPL tested. The neuronal latency was quantified using the peri‐stimulus time histogram (PSTH) of a neuron, by measuring the time from stimulus onset to an increase of neuronal activity above spontaneous activity (50 ms before stimulus presentation). Response duration was also calculated from the PSTH as the time stimulus‐evoked neuronal activity was above the level of spontaneous activity. Statistical testing was done with a Matlab two‐sided two‐sample *t*‐test (“*t*‐test2”) for normally distributed data, or a Matlab two‐sided Wilcoxon rank sum test for independent samples (“ranksum”) for not‐normally distributed data. For comparing more than two data sets, a one‐way ANOVA (Matlab “anova1”) with a correction for multiple testing (Tukey's honestly significant difference procedure). For all statistical tests, the significance level was set at *p*<0.05, if not stated otherwise. Mean values are presented with ± standard deviation values.

#### Response selectivity to echolocation and communication calls quantified by response strength

In a first step, we analyzed if units in the FAF and dAC responded selectively to call categories or single calls within each category by means of response strength. Following the method described in Ref. 15, we calculated the maximum number of spikes per bin evoked by either each call or call category. In detail, the PSTH (2 ms bin‐width) evoked by the 20 presentations of the calls from the three categories was extracted in a window set to match the longest response duration evoked by one of the 15 calls included in the three categories. Start and end of the response window was defined by the time in the PSTH where the response was stronger than the prestimulus activity recorded in the first 50 ms preceding the call presentation. A category preference index (PIcat) was computed as the number of categories in which the response summed over all calls within this category was at least 50% of the maximum evoked by the summed response in all categories. The PIcat could, therefore, have values between 1 (highest selectivity) and 3 (no selectivity). To quantify response selectivity for single calls within each category, a call preference index (PIcall) was calculated separately for the three categories. It was computed as the number of calls within a category for which the response was at least 50% of the maximum response per bin evoked by a call within a category. The PIcall could, therefore, have values between 1 (highest selectivity) and 5 (no selectivity).

#### Response selectivity quantified by the overall response pattern (confusion matrices)

The above‐described PIs only quantify if a unit is selectively responding to a certain call category or call within a category by means of the response strength. However, although calls from different categories might evoke the same number of spikes per presentation in a unit, the temporal response pattern might be different for the two types of calls. To take this into account and to test how specific units in the FAF and dAC responded to calls from the three categories, we constructed confusion matrices.

A confusion matrix quantifies the probability by which the neural response to a call can be specifically assigned to one specific call or call category (e.g., Ref. 34). This is based on the reproducibility of the spike response pattern evoked by a call or call category; if reproducibility is high for repeated call presentation, the response pattern can be correctly assigned to the corresponding call or call category. Otherwise, assignments will be random.

In detail, the PSTH (1 ms bin‐width) evoked by one of the 20 presentations of a single call (the test call) was extracted in a window set to match the longest response duration evoked by one of the 15 calls. Start and end of the response window was defined by the time in the PSTH where the response was stronger than the prestimulus activity recorded in the first 50 ms preceding the call presentation. This PSTH was compared to PSTHs in the same window randomly drawn from all other call presentations and the PSTH evoked by the same call by a different presentation within the 20 repetitions (to avoid that the spike sequence was compared to itself). The similarity of two spike patterns was quantified as the Euclidian distance, and the test call was assigned to that call category for which PSTH comparison resulted in the smallest Euclidian distance. If the PSTH comparison yielded two or more equal values for the Euclidean distance from different call categories, the PSTH was randomly assigned to one of those categories (forced choice). This procedure was repeated 10,000 times and all category assignments were collected, summed up, and the probability of correct assignments was calculated. If assignments were made to the correct category (i.e., the category of the test call), probability values would be highest along the diagonal line of the confusion matrix.

It is to be expected that the probability of correct assignments to the different categories will also depend on the temporal binning of the spike pattern (i.e., the temporal resolution with which the pattern is looked at). Therefore, we convolved the PSTH with a Gaussian window of 2‐ or 200‐ms duration and compared the probabilities of correct assignments for both temporal resolutions. To analyze if the correct classification of neural responses evoked by the test call were achieved for only one call category or for more than one, we calculated a selectivity index (SI) from the probability for correct assignments:

SI = maximum probability value—(mean + standard deviation of remaining values)

The SI takes into account both the absolute probability of the assignments as well the relative difference of the probability of assignments between all call categories. The highest possible SI value is 1. In addition to the confusion matrices for call category classification, we also constructed confusion matrices from the assignments of a response to the five calls within each category (within‐category classification).

## RESULTS

We recorded activity in response to acoustic stimulation (pure tones and/or prerecorded vocalizations) from 92 units in a locally confined region of the frontal cortex (the FAF) in both hemispheres of four anesthetized *P. discolor* (three females, one male). In detail, 23 out of 92 units (25%) in the FAF were recorded from the right hemisphere. To initially locate the FAF, the anterior sulcus was used as a landmark. Neurons responsive to acoustic stimulation were located in the region around this sulcus. Thereafter, stereotaxic coordinates guided the electrode penetrations. The position of the FAF in *P. discolor* roughly corresponds to the area in which the FAF is located in other bat species (e.g., *P. parnellii*,^8^ *C. perspicillata*,^11^, ^12^ *T. brasiliensis* ^15^). A frontal section showing the location of the FAF is presented in Figure S2.

**FIGURE 2:**
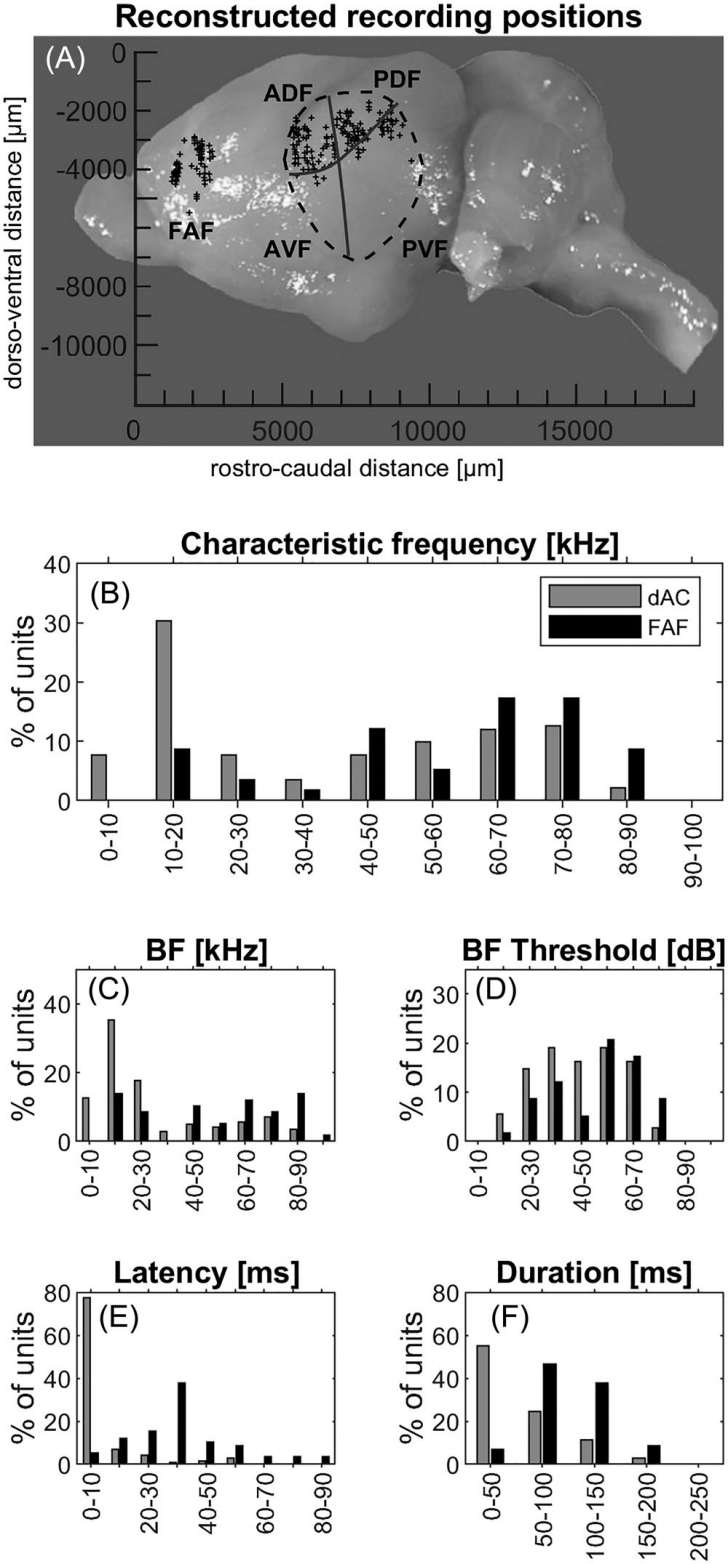
Recording sites and basic response properties. (A) Recording sites in the frontal auditory field (FAF) and the dorsal auditory cortex (dAC) of *P. discolor*. The dAC is defined as the anterior‐dorsal field and the posterior‐dorsal field of the auditory cortex. The total number of units was 92 in the FAF and 142 in the dAC. (B–F) Basic response properties of FAF and dAC units to pure‐tone stimulation. Abbreviators: ADF, anterior dorsal field; AVF, anterior ventral field; BF, best frequency; PDF, posterior dorsal field; PVF, posterior ventral field.

Recordings from 142 units in the *P. discolor* dAC (from both hemispheres of four anesthetized bats; three females, one male) stem from the data set published by Hörpel and Firzlaff,^22^ but none of the analyses or results presented here are part of this former publication. In detail, 30 out of 142 units (21%) in the dAC were recorded from the right hemisphere. The localization of recording sites in the FAF and the dAC are shown in Figure 2A.

### General response properties of FAF and dAC units

#### Frequency tuning

Representative examples of frequency–response areas (FRAs) from the FAF and the dAC, and their corresponding PSTHs, are shown in Figure S3. Of the dAC units, 94.4% (134/142) showed significant responses to stimulation with pure tones. In comparison, only 63.0% (58/92) of FAF units showed significant responses to stimulation with pure tones. In addition, 16.3% (15/92) of FAF units were only weakly responsive to pure‐tone stimuli and showed a patchy response pattern, that is, no consistent frequency‐response area was observed, but single frequency and SPL combinations did evoke responses (see Figure S3C).

The distribution of characteristic frequencies (CFs, frequency at which a given unit responds to the lowest sound intensity) in the FAF and the dAC is shown in Figure 2B. In the dAC, 38% (54/142) of units showed low CFs below 20 kHz, while only a few FAF units responsive to pure tones (14%, 8/58) had a CF this low and most had a CF between 40 and 90 kHz. Consequently, the mean CF of the FAF units was 56.74 kHz ± 20.7 kHz, which was significantly higher than the mean CF of the dAC units, which had a mean CF of 36.2 kHz ± 24.5 kHz (*t*(174) = 4.42, *p*<0.001). The sharpness of tuning of FRA measured as Q10dB was generally low in the FAF and the dAC (mean Q10dB: 4.4 ± 2.7 and 3.8 ± 2.7, respectively) and showed no significant difference (*t*(174) = 1.27, *p* = 0.2), although the distribution of Q10dB values in the dAC showed a higher proportion of low values (Figure S4).

The distributions of best frequencies (BFs, frequency with the strongest neuronal response) and BF threshold values in the FAF and dAC are shown in Figure 2C,D, respectively. Mean BF of the FAF units (51.8 kHz ± 25.7 kHz with a threshold of 49.4 dB ± 17.7 dB) was also significantly higher (*t*(174) = 5.42, *p*<0.001) than the results from the dAC units, which had a mean BF of 27.6 kHz ± 22.9 kHz with a threshold of 40.4 dB ± 16.0 dB. Thresholds were significantly lower in the dAC than in the FAF (*t*(174) = 2.28, *p*<0.05).

#### Response latency and duration

Most units in the dAC had short latencies up to 10 ms, whereas the latency distribution for the FAF units peaked around 30−40 ms (Figure 2E). As the latencies in the dAC show a non‐normal distribution, we show median and 25th and 75th percentile values. Median response latency of FAF units was 35.0 ms (27.0/74.1 ms), whereas dAC units showed a significantly shorter median response latency of 5.8 ms (4.6/8.2 ms; Wilcoxon rank sum test, *z* = 9.38, *p*<0.001). FAF units typically showed long, tonic responses to acoustic stimulation even to short pure tones (Figure 2F). Mean response duration was 94.8 ms ± 36.4 ms ranging from 41 to 192 ms. In contrast, units in the dAC showed significantly shorter responses with a mean response duration of 48.5 ms ± 45 ms ranging from 7 to 187 ms (*t*(189) = 6.42, *p*<0.001).

### Selectivity quantified by response strength

**FIGURE 3:**
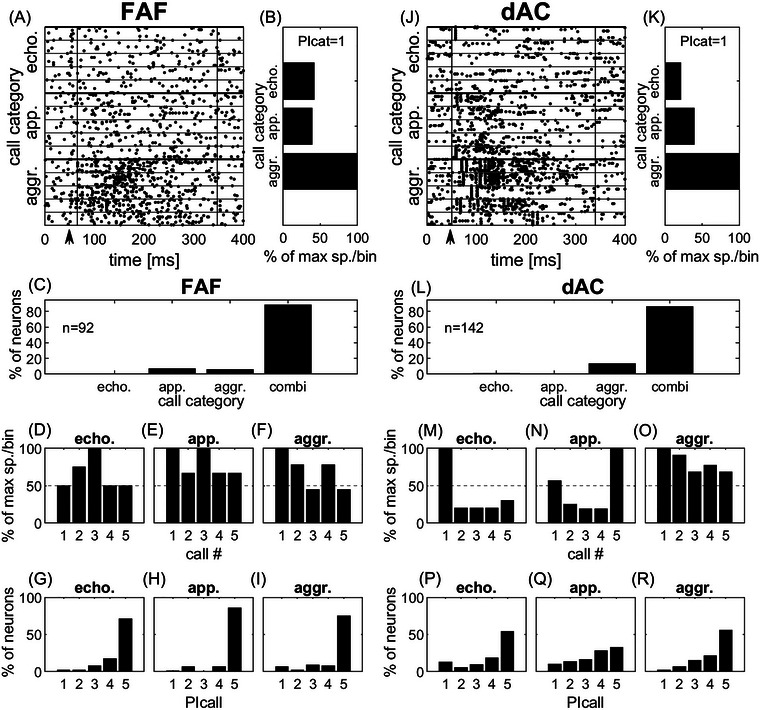
Selectivity quantified by response strength. (A–I) and (J–R) Responses of an FAF and a dAC unit, respectively, to calls of the three categories. (A, J) Spike responses are shown as raster plots. (B, K) Quantification of response strength (normalized to 100%) for the summed maximum response per 2‐ms bins for categories. Beginning and end of the analysis window for spike response quantification are shown by vertical black lines in (A) and (J). Stimulus onset was at 50 ms (black arrow). (C, L) Percentage of units selective for only one call category (PIcat = 1) or combination of categories (PIcat = 2 or 3) in the FAF and the dAC, respectively. (D–F) and (M–O) Quantification of response strength (normalized to 100%) for the summed maximum response per 2‐ms bin of individual calls within the categories for the FAF unit shown in (A), and the dAC unit shown in (J), respectively. (G–I, P–R) Summary‐plots of call‐preference (PIcall) of all units for single calls within the three call categories in the FAF and the dAC, respectively. The total number of units was 92 in the FAF and 142 in the dAC. Abbreviations: aggr., aggression; app., appeasement; combi, combinations of two or three categories; dAC, dorsal auditory cortex; echo., echolocation; FAF, frontal auditory field.

Next, we investigated the response of the FAF and dAC to call categories to determine if these regions were selective for calls with different functions (echolocation vs. communication) or social valence (appeasement, aggression) from the *P. discolor* repertoire. In addition, we investigated if units could encode for individual calls within these categories or if responses to all calls within a category were the same. Figure 3A shows an example of a raster plot of an FAF units response to the three call categories. The normalized maximum response for this unit per 2 ms bins summed up over all five calls within a category (Figure 3B) reveals that aggression is the best responded call category, while the summed responses for the echolocation call and appeasement call category each make up less than 50% of the response evoked by the aggression call category. An example of a unit from the dAC (Figure 3J,K) shows the same response behavior: the summed responses evoked by a call from the aggression call category are stronger, while the response strength is less than 50% in the two other categories. Therefore, these examples of FAF and dAC units were selectively responding to only one call category (PIcat = 1, see Methods).

However, when looking at all units in both regions, it was clear that only a small proportion of units were selectively responding to calls from a single category (Figure 3C,L). In the FAF, 88% (81/92) of units responded to calls from two or three categories (“combi”), while only 12% (11/92) of units selectively responded to calls from only one category (Figure 3C). In detail, 7% (6/92) were selective for appeasement calls and 5% (5/92) were selective for aggression calls. None of the 92 units recorded in the FAF selectively responded to echolocation calls. In the dAC, only slightly more (14%, 20/142) units selectively responded to calls from only one category (Figure 3L). In detail, 13% (19/142) of units were selective for the aggression call category, while only 1% (1/142) of units were selective for the echolocation call category. The remaining 86% (122/142) of units responded to calls from two or three categories (“combi”).

When looking at the response selectivity for single calls within each of the different categories (echolocation, appeasement, aggression), the results show that the majority of FAF units did not selectively respond to single calls within the three categories. This is exemplified in Figures 3D–F for the unit shown in Figure 3A. The PIcall is at least 3 for each of the categories, that is, at least three calls evoked a response of at least 50% of the maximum spikes per 2 ms bin. In summary, in the FAF, 71% (65/92), 86% (79/92), and 74% (68/92) of units responded to all of the five calls (PIcall = 5) within the echolocation, appeasement, and aggression call category, respectively (Figures 3G–I).

Selectivity for single calls quantified by response strength was higher in the dAC than in the FAF. Although for the dAC unit shown in Figure 3J, the PIcall for the aggression category was 5 (Figure 3O), the two other categories had a PIcall of 1 (echolocation call category) and 2 (appeasement call category; Figure 3M,N). Note that a PIcall of 1 indicates the highest preference, that is, the unit was selective for one call within a category (see Methods). In summary, in the dAC, only 54% (77/142), 32% (46/142), and 56% (79/142) of units had a PIcall value of five (Figures 3P–R) for the three different call categories, respectively. The higher within‐call category selectivity is also illustrated by the number of units selectively responding to only one call within a call category (PIcall = 1), especially when looking at the echolocation and appeasement call categories. While in the dAC, 13% (18/142) and 10% (14/142) of units showed a PIcall of 1 for echolocation calls and appeasement calls, respectively (Figure 3P,Q), in the FAF, only 2% (2/92) and 1% (1/92) of units had a PIcall of 1 for these types of calls (Figure 3G,H). In contrast, only in the aggression call category, the number of units with a PIcall of 1 was higher in the FAF (7%, 6/92) than in the dAC (2%, 3/142, Figure 3I,R). In summary, selectivity quantified by response strength was slightly higher in the dAC than in the FAF, both for call category and within‐category comparison.

### Confusion matrices: Call category classification based on spike patterns

**FIGURE 4:**
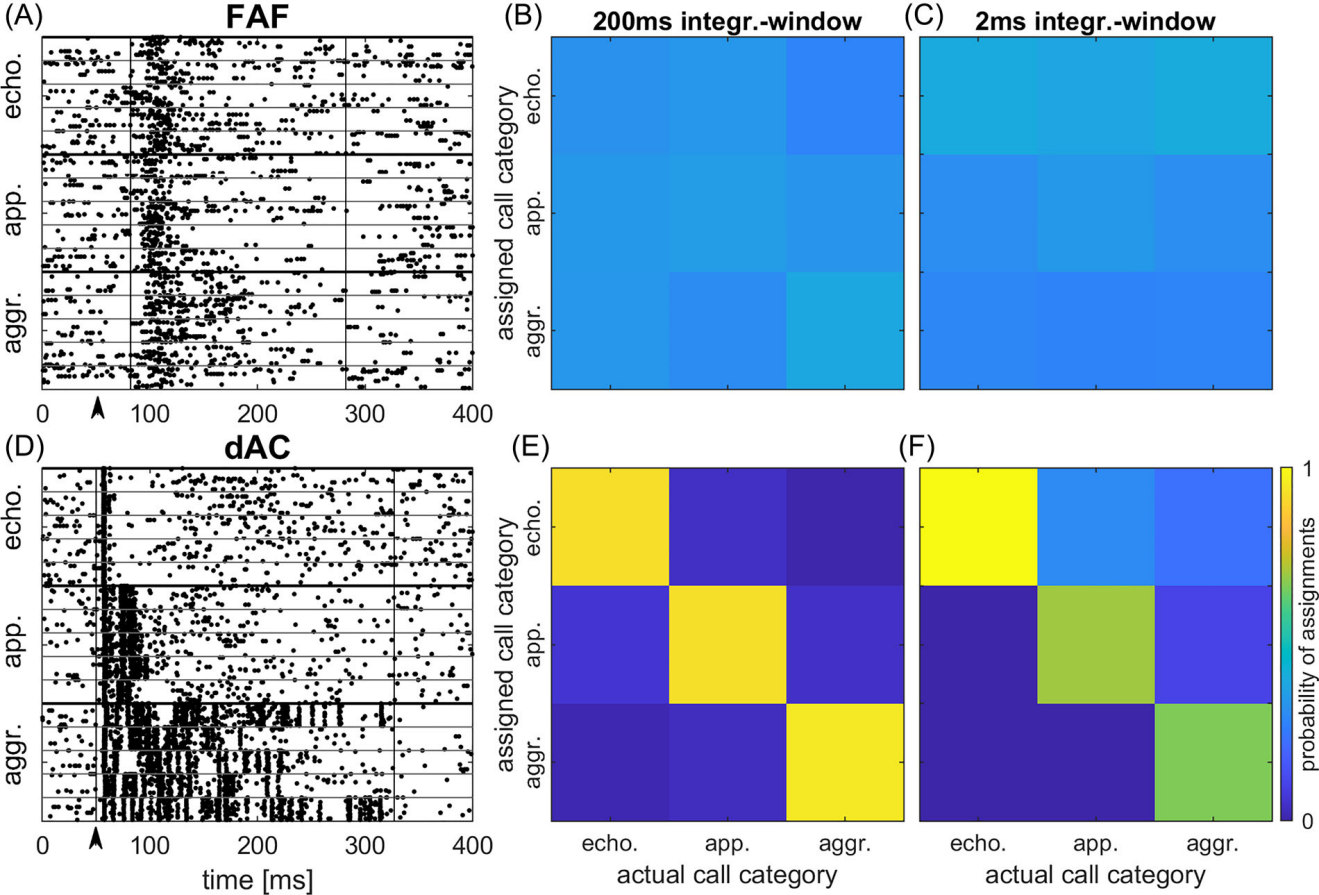
Spike response patterns and confusion matrix for category assignments. (A–C) Response pattern evoked by the 15 prerecorded calls from the three categories in an FAF unit and confusion matrices after convolution of the spike pattern with a 200‐ and 2‐ms integration window. (D–F) Same for a dAC unit. Beginning and end of the analysis window for spike response quantification are shown by vertical black lines in (A) and (D). Stimulus onset was at 50 ms (black arrow). FAF unit (A): CF = 78 kHz, BF = 85 kHz; dAC unit (D): CF = 48 kHz, BF = 17 kHz. Abbreviations: aggr., aggression; app., appeasement; dAC, dorsal auditory cortex; echo., echolocation; FAF, frontal auditory field, integr., integration.

The above‐described neuronal selectivity was based on response strength. Next, we assessed the temporal responses since although calls from different categories might evoke the same number of spikes per presentation in a unit, their temporal response pattern may still be different for the two types of calls. Call types could then be discriminated based on the temporal response pattern, while this would not be possible based on the mere spike count. Figure 4A shows the responses of a unit in the FAF to calls of the three categories. Each of the five calls within the three call categories evoked a strong tonic response. These patterns were not distinctive for a given call type and consequently, the probability by which the response to a call could be assigned to a call category is low in the confusion matrix (Figure 4B, 200‐ms integration window for PSTH convolution, see Methods). By contrast, the example of the dAC unit (Figure 4D) shows a distinctive response pattern for each of the three call categories, both in terms of response duration and temporal spike pattern. Therefore, the confusion matrix (Figure 4E) displays very high probability values (>90%) for category‐specific assignment of responses along the diagonal, that is, the spike response pattern evoked by a single call presentation could be assigned to the correct category in most cases and with almost similar probability. Using a shorter (2‐ms) integration window had no effect for the FAF unit call assignment probability (Figure 4C). Probabilities of assignments to the categories were still low. For the dAC unit, correct assignment can still be made with high probability using a shorter integration window (Figure 4F). However, the probability of correct assignment for the aggression and the appeasement categories is lower as for the confusion matrix was constructed using a 200‐ms integration window, while the probability of correct assignment for the echolocation category had even increased.

**FIGURE 5:**
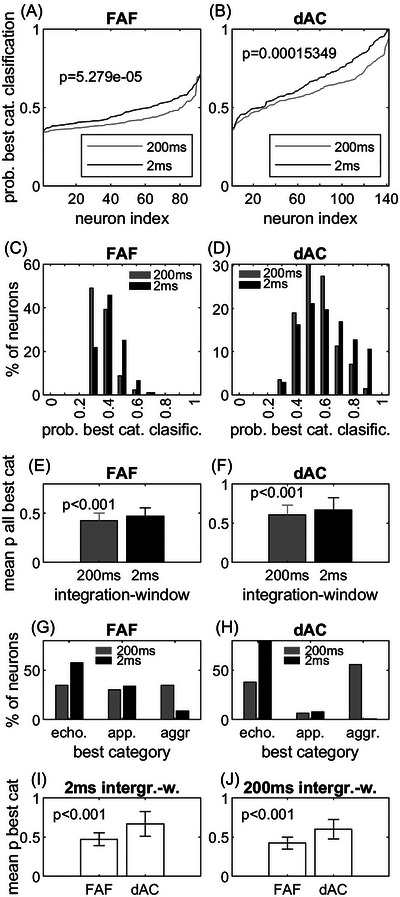
Quantification of best category assignments in confusion matrices for FAF and dAC units. (A, B) Values for probability of best assignments to a category in the FAF and the dAC compared for the 200‐ms (gray line) and the 2‐ms (black line) integration window. (C, D) Histogram distribution of the values shown in (A) and (B). (E, F) Comparison of the mean probability values for best category assignment for the 200‐ms (gray bars) and the 2‐ms (black bars) integration window in the FAF and dAC, respectively. (G, H) Comparison of percentage of units showing best assignment for one of the three call categories for 200‐ms (gray bars) and 2‐ms (black bars) integration window in the FAF and dAC, respectively. (I, J) Comparison of the mean probability values for best category assignment in the FAF and the dAC for the 2‐and 200‐ms integration window, respectively. Abbreviations: aggr., aggression; app., appeasement; cat., category; dAC, dorsal auditory cortex; echo., echolocation; FAF, frontal auditory field, integr.‐w., integration window; prob., probability.

Figure 5 summarizes the responses from all units in the FAF and dAC. In both the FAF and dAC, the maximum values for the probability of correct category classification from the confusion matrices (i.e., the probability values for the best category) were significantly higher for the 2‐ms integration window than for the 200‐ms integration window (Figure 5A,B; *t*‐test, *p*<0.001 for both regions, FAF: *t*(182) = −4.14, *p*<0.001 and dAC: *t*(282) = −3.83, *p*<0.001). This is also reflected in the histograms for the probability of best category classification (Figure 5C,D). The histogram distribution shifts to the right toward higher probability values in both regions when a 2‐ms integration window was used. These differences were statistically significant when comparing the values for best probabilities for correct classification for 2‐ and 200‐ms integration windows in the FAF and the dAC (Figure 5E,F, *t*‐test, *p*<0.001 for both the FAF (*t*(182) = −4.14, *p*<0.001) and dAC (*t*(282) = −3.83, *p*<0.001). As can be seen from Figure 4E,F, the length of the integration window (i.e., the temporal integration of response pattern) could have an influence on the assignment of response to the three different call categories. When analyzing this in more detail, the results show that for the 200‐ms integration window best probability of correct classification values in FAF units were equally distributed over the three call categories. However, there was a preference (>50% of units) for echolocation calls when a 2‐ms integration window was applied (Figure 5G). For dAC units, differences were even more pronounced: most units (>50%) show the best probability of correct classification values for the aggression calls using a 200‐ms integration window, while most units show best classification values for echolocation calls using a 2‐ms integration window (Figure 5H).

So far, we have looked at the influence of the temporal integration window on the probability of correct classification of neural responses to the three call categories. However, Figure 5A,B also indicates that the values for best category classification are higher in the dAC than in the FAF. Quantified in more detail for both the 2‐ and 200‐ms integration window conditions, the values for best category classification are significantly higher for the 2‐ms window (*t*(232) = 10.83, *p* <0.001) and for the 200‐ms window (*t*(232) = 12.19, *p*<0.001) in the dAC than in the FAF (Figure 5I,J). In summary, units in the dAC can more reliably classify calls from different categories than units in the FAF. In both cortical areas, classification performance is best when the temporal spike pattern is analyzed with a high temporal resolution.

### Confusion matrices: Within‐category classification based on spike pattern

Although a unit could show a high probability of correct response assignments for one or more of the three call categories, it cannot automatically be inferred that response classification within a category can also be reliably done. The response patterns evoked by the single calls might be different for calls from the different categories, but relatively similar for calls from the same category. This is analyzed in detail in the following.

**FIGURE 6:**
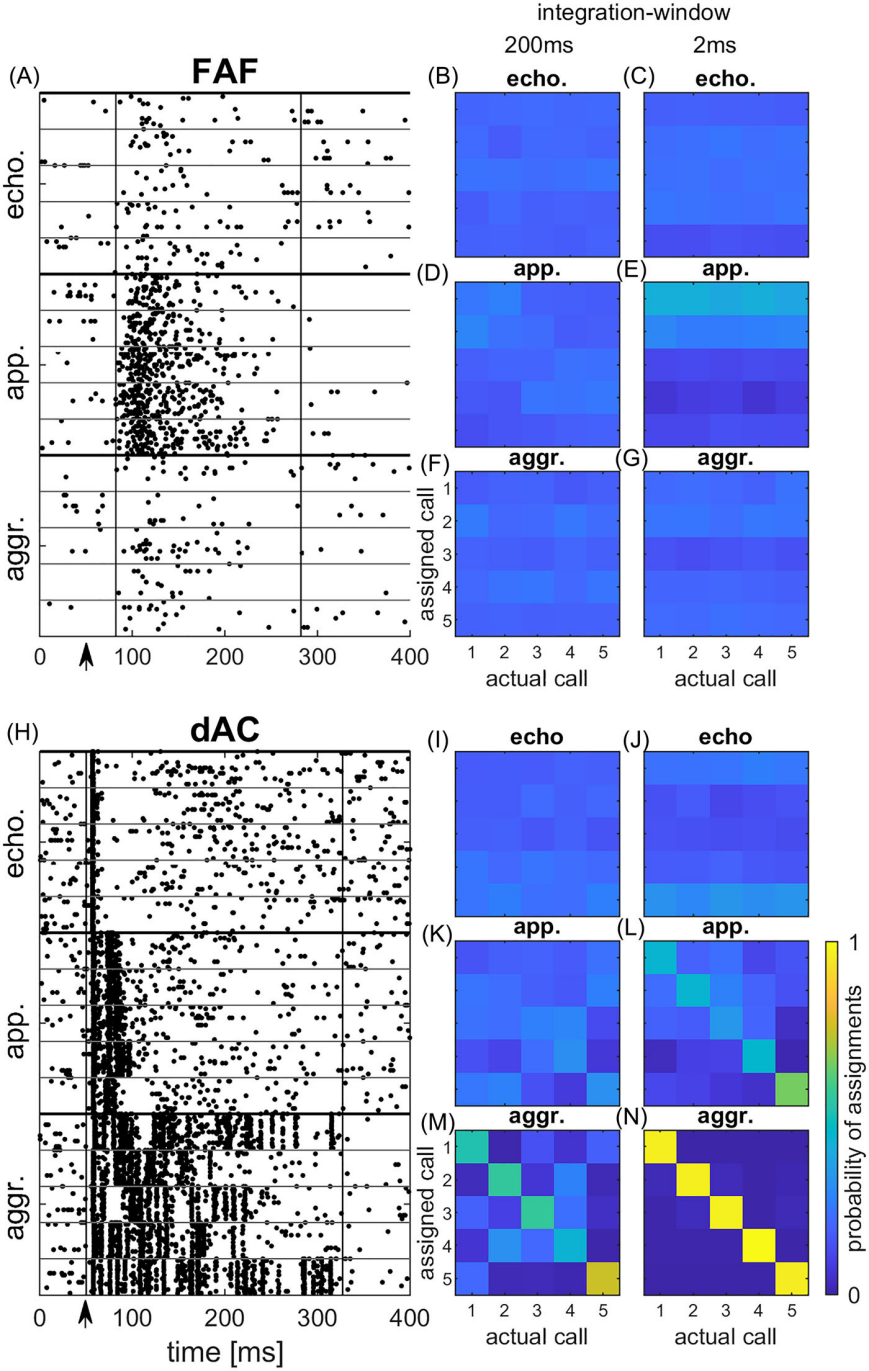
Spike response patterns and confusion matrix for within‐category call assignments in an FAF unit (A–G) and a dAC unit (H–N). (A) Response pattern evoked by the 15 prerecorded calls from the three categories in an FAF unit. (B, D, F) Confusion matrices of this unit for the five calls within each call category after convolution with a 200‐ms integration window. (C, E, G) Same for the 2‐ms integration window. (H) Response pattern evoked by the 15 prerecorded calls from the three categories in a dAC unit. Beginning and end of the analysis window for spike response quantification are shown by vertical black lines in the raster plot. Stimulus onset was at 50 ms (black arrow). (I, K, M) Confusion matrices of this unit for the five calls within each call category after convolution with a 200‐ms integration window. (J, L, N) Same for the 2‐ms integration window. Abbreviations: aggr., aggression; app., appeasement; cat., category; dAC, dorsal auditory cortex; echo., echolocation; FAF, frontal auditory field.

Figure 6A shows the spike raster plot and confusion matrices for within‐category classification for an FAF unit showing a strong response to the appeasement call category. Although for this unit the probability for correct assignment was high for the appeasement calls, at least for the 200‐ms integration window (Figure S5), within‐category probability for correct assignment were always low for both the 200‐ms (Figure 6B,D,F) and the 2‐ms integration window (Figure 6C,E,G). In other words, none of the five calls from each category evoked a distinguishable response pattern in this unit. In contrast, in the dAC unit (Figures 6H–N; previously shown in Figure 4D), within‐category classification probability was high for all five calls in the aggression category for the 2‐ms integration window (Figure 6N) and for one call for the 200‐ms integration window (Figure 6M). For the 2‐ms integration window, at least one appeasement call spike response pattern could be assigned correctly with high probability (Figure 6L).

**FIGURE 7:**
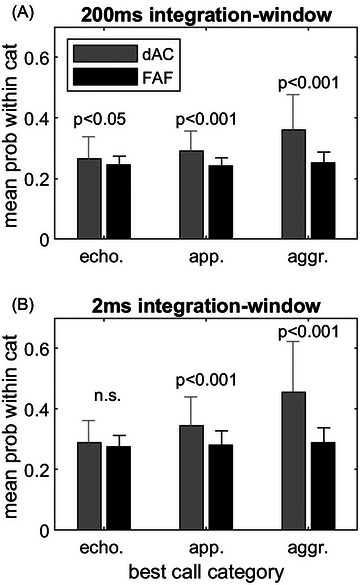
Comparison of the mean probability values for best within‐category assignment in the dAC and the FAF for the 200‐ms integration window (A) and the 2‐ms integration window (B). Abbreviations: aggr., aggression; app., appeasement; cat, category; dAC, dorsal auditory cortex; echo., echolocation; FAF, frontal auditory field; n.s., not significant; prob, probability.

We compared the values for best category classification for the responses evoked by each of the five single calls from each category for units in the dAC and the FAF (Figure 7). For both integration windows, the mean of the values for best classification within a category in the dAC was lowest for the echolocation calls, intermediate for the appeasement calls, and highest for the aggression calls (Figure 7A,B) for the 2‐ms (*F*(2,423) = 70.82, *p*<0.001) and *F*(2,423) = 43.33, *p*<0.001 for the 200‐ms integration window). For FAF units, the values were not significantly different across call categories (*F*(2,273) = 2.11, *p*≥0.05 for the 2 ms and, *F*(2,273) = 2.47, *p*≥0.05 for the 200‐ms integration window). For both integration windows, the values for best classification within a category were significantly higher for appeasement and aggression calls in the dAC than in the FAF for the 200‐ms window (appeasement: *t*(232) = 6.66, *p*<0.001; and aggression: (*t*(232) = 8.47, *p*<0.001) and for the 2‐ms window (appeasement: *t*(232) = 5.92, *p*<0.001; and aggression: *t*(232) = 9.18, *p*<0.001). For the echolocation call category, a significant difference was only present between dAC and FAF for the 200‐ms integration window (*t*(232) = 2.49, *p*<0.05). In summary, as already shown for category classification, within‐category classification of single calls is more reliable in the dAC than in the FAF.

### Confusion matrices: Call category and within‐category selectivity based on spike pattern

**FIGURE 8:**
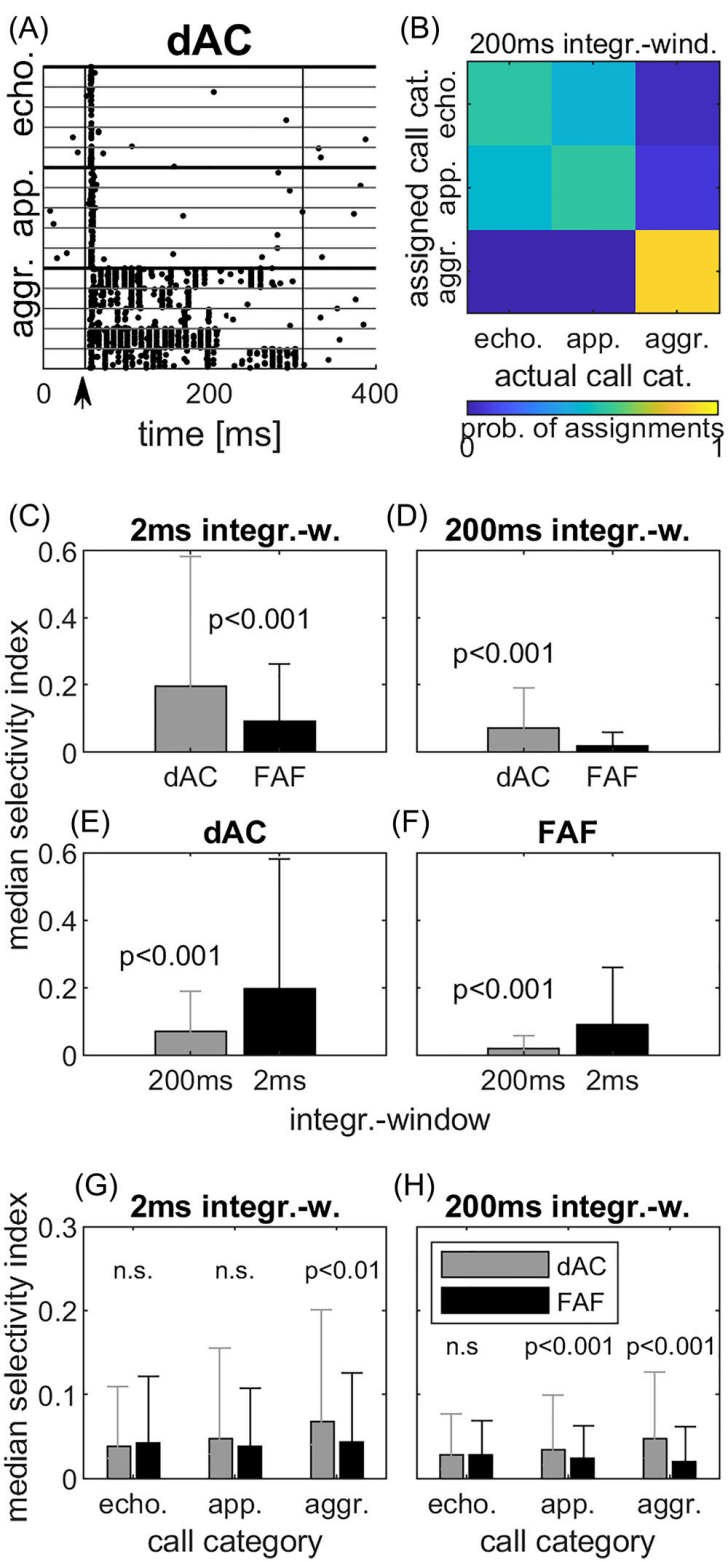
Selectivity indices (SIs) for call category confusion matrix assignments in FAF and dAC units. (A) Raster plot and (B) confusion matrix for category assignment of a dAC unit. Beginning and end of the analysis window for spike response quantification in (A) are shown by vertical black lines in the raster plot. Stimulus onset was at 50 ms (black arrow). The unit has an SI of 0.34 (see Methods for details on the calculation of the SI). (C, D) Comparison of the median SI for best category assignment in the dAC and the FAF for the 2‐ and 200‐ms integration window, respectively. (E, F) Comparison of the median SI for best category assignment for the 2‐ and 200‐ms integration window in the dAC and the FAF, respectively. (G, H) Comparison of the median SI for within‐category confusion matrix call assignments in the dAC and the FAF. (G) Median SI for calls within the three categories for the 2‐ms integration window and (H) the 200‐ms integration window. Abbreviations: aggr., aggression; app., appeasement; cat., category; dAC, dorsal auditory cortex; echo., echolocation; FAF, frontal auditory field; integr.‐w, integration window; n.s., not significant.

The SI shows if correct classifications of neural responses in the confusion matrices were achieved for only one call category or single calls within a category (high SI) or for more than one (low SI; see Methods for details on the calculation of the SI). Figure 8A,B, shows an example of a dAC unit with a relatively high SI of 0.34. While short echolocation calls as well as the longer appeasement calls evoked an onset response only, aggressions calls evoked a distinct but category‐specific spike pattern for each of the five calls (Figure 8A). Consequently, the probability of correct classification of neural responses was higher for the aggression call category than for the two other call categories (Figure 8B). The overall range of SI for call categories in the dAC was from −0.10 to 0.86 and −0.03 to 0.39 for 2‐ and 200‐ms integration windows, respectively (Figure 8C,D). The FAF ranged from −0.06 to 0.05 and −0.04 to 0.27 for 2‐ and 200‐ms integration windows, respectively. Comparing the SIs from units in the dAC and the FAF, we found that dAC units generally showed a higher selectivity for a certain call category than units in the FAF for both integration windows (Figure 8C,D, Wilcoxon rank sum test, *z* = 4.29, *p*<0.001 and *p*< 0.001, *z* = −5.24, *p*<0.001 for the 2‐and‐200 ms integration window, respectively). However, the integration window had a profound effect on the SI of units in both the dAC and FAF. Selectivity was always higher when a 2‐ms integration window was applied (Figure 8E,F, Wilcoxon rank sum test *z* = −6.48, *p*<0.001, and *z* = −5.98, *p*<0.001, respectively, for the dAC and the FAF).

When looking at within‐category call selectivity, SIs for aggression calls in the dAC were significantly higher than in the FAF for both integration windows (Figure 8G,H; Wilcoxon rank sum test, *z* = 3.12, *p*<0.01, and *z* = 5.61, *p*<0.001 for the 2‐ and 200‐ms window, respectively). In addition, SIs for appeasement calls were higher in the dAC for the 200‐ms integration window (Wilcoxon rank sum test, *z* = 3.48, *p*<0.001; Figure 8H), while echolocation calls showed the smallest SIs for both integration windows and showed no significant difference between the FAF and the dAC (Wilcoxon rank sum test, *z*‐value = −0.5724 *p*≥0.05, and *z* = 1.19, *p*≥ for the 2‐ and 200‐ms window, respectively; Figure 8G,H). To summarize, selectivity for call categories was generally higher in the dAC than in the FAF, with SIs being highest for responses evoked by aggression calls.

## DISCUSSION

The present study investigated the basic response properties of units in the FAF and the dAC of the *P. discolor* bat and compared neural responses to a library of 15 echolocation and communication calls in these regions. A special focus was put on comparing call selectivity across regions and asking if response patterns of FAF and dAC units allowed call classification individually or by category. Neuronal responses showed that dAC units are capable of high‐fidelity call selectivity, both in identifying call categories but also individual calls. By contrast, the performance of FAF units in these tasks was of lesser quality.

### Basic response properties

In the FAF, neuronal responses evoked by pure tone stimuli were characterized by long latencies and tonic response patterns. Furthermore, response variability was high, for example, first‐spike latencies, and the overall response pattern differed strongly throughout stimulus presentations. Frequency response areas often showed complex, patchy shapes. Thirty‐seven percent of FAF units were unresponsive to pure‐tone stimulation. Our results, therefore, support earlier findings in the FAF of other bats.^10^ ^−^ ^12^, ^15^ In contrast to the FAF responses, dAC units showed more precise phasic response pattern with shorter latencies and shorter durations. The large percentage of units with CF below 20 kHz in the dAC was somewhat unexpected as the dorsal fields of the AC (the anterior dorsal field [ADF] and the posterior dorsal field [PDF]) are considered to contain mainly neurons tuned to high frequencies.^21^ This might be due to a bias coming from using different stimuli to search for neuronal activity (pure tones in Ref. 21 and communication calls in the present study). In addition, some of the dAC units were located at the borders to the ventral fields, especially the posterior ventral field (PFV, see Figure 2A). We, therefore, might have picked up some units from the low‐frequency part of the tonotopic representation in this field.

### Call‐category or within‐category classification

The above‐described differences in the basic response properties also seem to be reflected in the differences seen in the probability for correct call‐category or within‐category classification of FAF and dAC units. As dAC units often responded with distinct spike patterns to echolocation and communication calls (in accordance with the phasic responses evoked by pure‐tone stimulation), single spike trains could be more often assigned correctly to the corresponding call category in the dAC than in the FAF. This effect was most prominent for the 2‐ms integration window which yielded higher probabilities of correct category classification than the 200‐ms integration window. While this observation is not surprising regarding the distinct temporal spike patterns often observed in the dAC, the finding that the 2‐ms temporal integration widow yielded higher classification probability for the call category in the FAF, too, is somewhat counterintuitive. This is because the tonic responses of FAF units typically lack a distinct temporal patterning that might facilitate classification when a short integration window is used. A relief from this inconsistency might be found when looking at Figure 5G. For the 2‐ms integration window, most units showed the highest probability of correct category classification for the short echolocation calls but not for the long aggression calls, in both the FAF and the dAC. Therefore, short responses evoked by echolocation calls might favor high response classification probabilities when the short 2‐ms integration window is applied. For long responses evoked by the longer aggression call, however, the 200‐ms integration window is most effective for category classification. The results for the within‐category classification can be interpreted in the same way; the distinct phasic spike pattern of dAC units facilitated the within‐category classification of one or more calls compared to the FAF, especially for short integration windows.

It has been shown that communication calls are analyzed by *P. discolor* based on short temporal integration times.^35^ In addition, Schnupp et al.^34^ showed that neurons in A1 of ferrets efficiently represented vocalizations through temporal spike pattern codes when analyzed at short timescales of 10−50 ms. The fact that temporally integrating calls in 2‐ms intervals yielded higher probabilities of correct classification than for the 200‐ms integration windows in *P. discolor* is in line with these earlier findings. However, it is not clear if the increased accuracy over short integration times is due to physiological mechanisms in the neocortex such as membrane voltage fluctuations in the gamma frequency band (20–70 Hz, i.e., at periods of 14−50 ms^36^) as suggested by Schnupp et al.^34^ Short integration times may simply be favored by the distinct pattern of spike response peaks caused by envelope fluctuations found especially in the aggression calls of *P. discolor*. This notion is supported by the inter‐ and within‐category variability of call duration and temporal envelope for these calls (see Figure S1).

### Call‐category or within‐category selectivity

In our experiments, we quantified selectivity by response strength or by the probability of correct classification. In both cases, units in the dAC and the FAF typically did not show high selectivity. Few units in both areas were selective for only one of the three call categories or single calls within these categories. However, whereas no differences were observed for the selectivity for a certain category based on response strength (PIcat), units in the dAC showed significantly higher SIs for call category classification based on the probability of correct classification (see Figure 8) than units in the FAF. For within‐category classification, the dAC units were more selective for single calls than FAF units, both when looking at the response strength (PIcall) as well as for selectivity indices on the probability of correct classification based on spike patterns. As already discussed above, one can assume that these differences can be attributed to the ability of dAC units to encode call envelopes more precisely because of their phasic response pattern and their ability to follow fast amplitude modulations^22^ than FAF units and, probably, A1 neurons, too. Therefore, dAC units may also translate differences in call envelop into larger differences of overall response strength than neurons in A1 and, therefore, might show a slightly stronger preference for certain single calls.

### Functional relevance of our findings

Based on our results, it is difficult to speculate on the precise functional role of the dAC and the FAF in the frontal cortico‐striatal network for vocal–motor control. Although the localization of the FAF in the *P. discolor* cortex has been described,^17^ it is not fully clear if additional frontal areas exist that might also receive auditory input. It, therefore, might well be that our recordings did not cover responses from neurons of all cortical regions relevant to this network. For example, Gooler and O'Neill^37^ reported that communication calls could be elicited by microstimulation from a region in the anterior cingulate cortex in the bat (*P. parnellii*). Interestingly, the anterior‐most cingulate cortex shows significant functional connectivity with dorsal regions of the left AC in *P. discolor*,^38^ which was not observed for the right hemisphere. This would be in line with earlier findings in other bats, which hint toward a preference for the processing of social calls in the left hemisphere.^39^ As the number of units recorded from was not evenly distributed across both sides in the FAF and the dAC in our study, we did not report here on possible hemispheric specializations for the processing of communication sounds.

However, the findings of our study imply that units in the dAC encode call category as well as single calls from the same category of behavioral context more reliably than units in the FAF. For example, the dAC unit shown in Figure 4 was responsive to all calls from all three categories; however, the response pattern allowed for precise classification of call categories as well as classification of responses to individual calls, at least for the aggression call category (see Figure 6N). This type of unit could, therefore, play a role in the identification of vocalizations and might be important for feedback control (i.e., template matching) during vocal learning.^40^ Other units in the dAC were more selective at encoding a single category of calls, and, therefore, might represent a second stage of more generalized processing of auditory information. Interestingly, call category‐selective neurons are already present in the inferior colliculus of bats,^41^ and social context enhances neuronal population responses to social vocalizations in that area.^42^ Thus, the organization of call meaning already seems to start on subcortical levels. In the FAF, a response behavior as seen in the units of the dAC was less frequently observed in our study. Call selectivity was almost absent and call category selective units were rare, too. This was mainly due to the tonic response patterns, which lacked precise coding of the spectro‐temporal envelopes of vocalizations. However, at least some units in the FAF could selectively code for a certain call category although within‐category classification of a single call was not possible based on the spike response pattern or spike response strength (see raster plot of the FAF unit shown in Figure 6A). The confusion matrix for call category classification based on spike patterns for that unit is shown in Figure S5. This type of response behavior of FAF units would support the notion that higher processing stages become progressively more tolerant of variation of calls carrying the same meaning.

It should be noted that our findings that dAC units were more selective at encoding call category as well as individual call types than FAF units in *P. discolor* is opposite to the results obtained in the Mexican free‐tailed bat by Macias et al.,^15^ who found the FAF to be more selective than A1. This suggests that the processing of acoustic features related to echolocation and communication calls might be different in the different fields of the AC. The dorsal cortical fields (the ADF and the PDF in *P. discolor* ^21^) are known to feature units capable of fast and precise temporal processing for coding of echo‐delay as well as for coding envelope fluctuations of complex stimuli such as communication calls.^22^, ^43^ Therefore, the dAC might be preadapted to serve a role for encoding call meaning within a cortico‐striatal network.

It is difficult to say how the transition from precise response patterns from the dAC to the more “noisy” pattern in the FAF occurs. The FAF receives input not just from the AC but also via the extra‐lemniscal pathway^8^ bypassing the AC. López‐Jury et al.^12^ suggested that slow, subthreshold, synaptic dynamics are underlying the long‐lasting, less precise spiking pattern of neurons in the FAF of bats. It remains unclear how the FAF can fulfill a role on vocal–motor control in communication and even more so in echolocation, which requires fast and precise motor actions to adjust ear movement and fast call repetition rate. It is likely that anesthesia is having an influence on spiking pattern in the FAF, as at least for FM sweeps, response duration was shorter and, therefore, more precise in the FAF than in the A1 in awake bats.^15^ It is also possible that the precision of spike response in the FAF could increase when bats are engaged in a behavioral‐relevant task. On the other hand, it can be speculated that long and noisy responses might serve sensory motor integration as sensory input of different modalities that can be integrated over time, as discussed by López‐Jury et al.^12^

## CONCLUSION

In conclusion, the data presented here support the notion that the dAC plays an important role in the neural network for processing and control of vocal communication in bats. The precise timing of responses encode temporal envelope properties of individual calls as well as call categories, and, therefore, precise information about the auditory input in a vocal communication context could be relayed upstream in the cortico‐striatal network.

## AUTHOR CONTRIBUTIONS

S.G.H. and U.F. conceived and designed the research; S.G.H. performed experiments; S.G.H. and U.F. analyzed data; S.G.H. and U.F. interpreted results of experiments; U.F. prepared figures; U.F. and S.G.H. drafted the manuscript; S.G.H., S.C.V., and U.F. edited and revised the manuscript; U.F. and S.C.V. approved the final version of the manuscript.

## FUNDING

This work was supported by Human Frontier Science Program Grant RGP0058 to U.F. S.C.V. was supported by a UKRI Future Leaders Fellowship (MR/T021985/1), and S.C.V. and S.G.H. were supported by an ERC Consolidator Grant (101001702; BATSPEAK) awarded to S.C.V.

## COMPETING INTERESTS

No conflicts of interest, financial or otherwise, are declared by the authors.

## Supporting information



Figure S1 Swarm chart showing the inter and inner‐call‐category variability of four acoustic parameters.

Figure S2 Location of the FAF *in P. discolor*.

Figure S3 Examples of frequency response areas (FRAs) from three units in the FAF (A−C) and the dAC (G−I) and their corresponding PSTHs (D−F and J−L, respectively).

Figure S4 Distribution of Q10 dB values of frequency response areas of FAF and dAC units.

Figure S5 Spike response patterns and confusion matrix for category assignments for the FAF unit shown in Figure 6A.

## Data Availability

Research data are not shared.
