# Supplementary figures and images for: Representation of vocalizations in the frontal auditory field and the dorsal auditory cortex of bats
Source: Ann N Y Acad Sci. 2025 Apr 8;1547(1):116–30. doi: 10.1111/nyas.15336 (PMC12096809; doi:10.1111/nyas.15336)

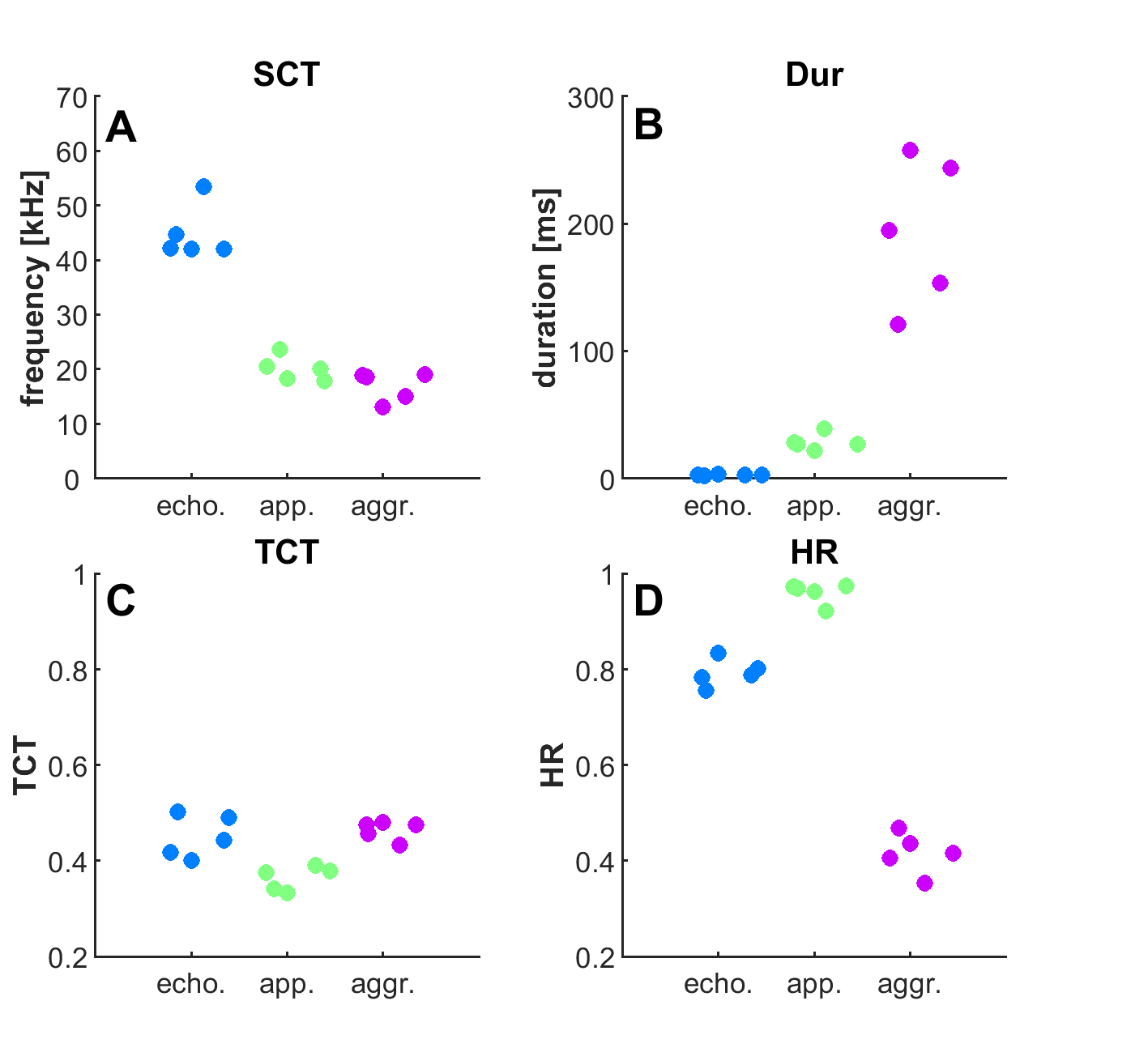

Supplement: Supplementary file 1 — Figure S1 Swarm chart showing the inter and inner‐call‐category variability of four acoustic parameters. [file NYAS-1547-116-s003.tif]

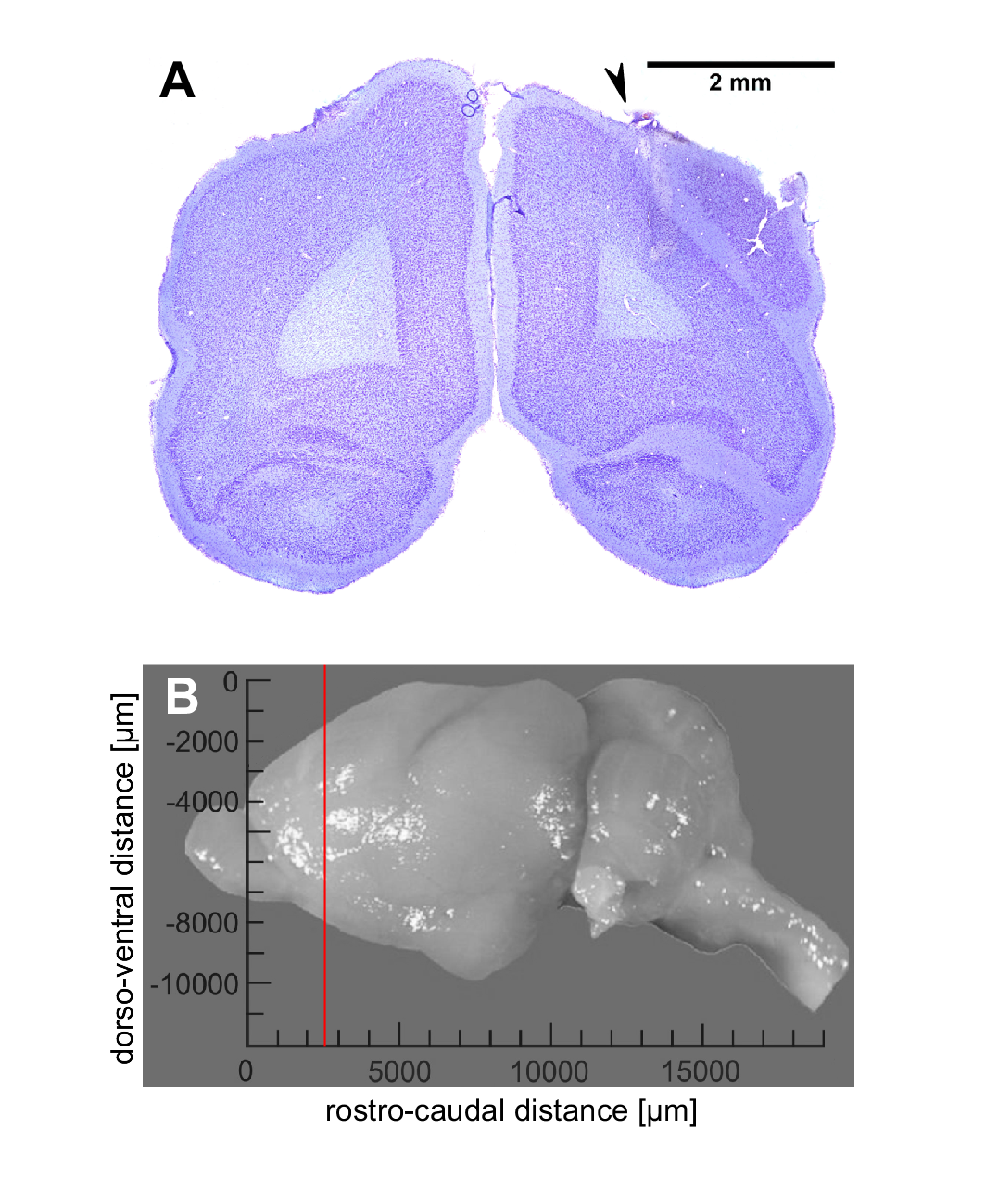

Supplement: Supplementary file 2 — Figure S2 Location of the FAF in P. discolor. [file NYAS-1547-116-s002.tif]

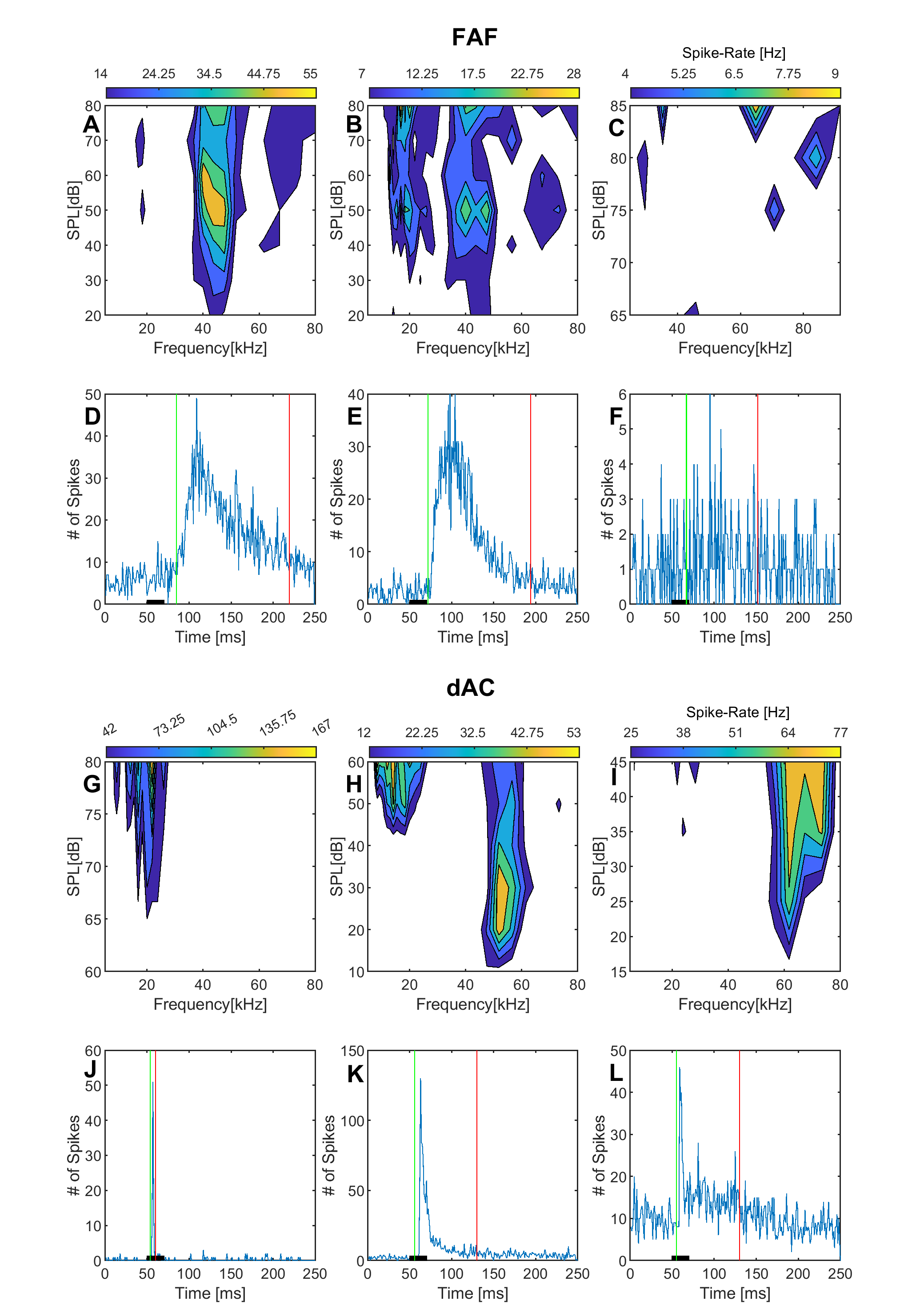

Supplement: Supplementary file 3 — Figure S3 Examples of frequency response areas (FRAs) from three units in the FAF (A−C) and the dAC (G−I) and their corresponding PSTHs (D−F and J−L, respectively). [file NYAS-1547-116-s001.tif]

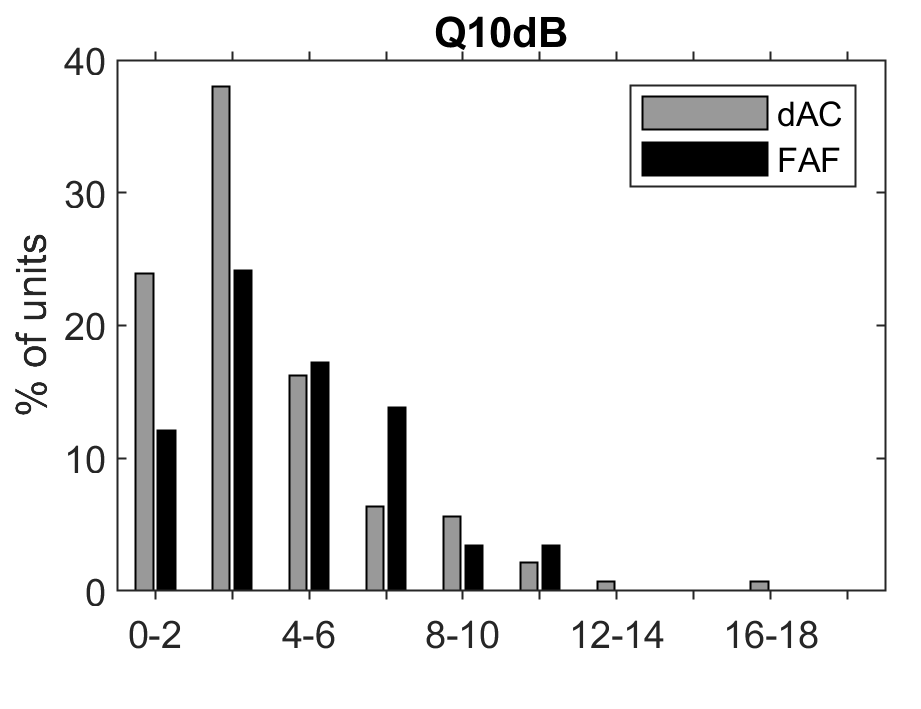

Supplement: Supplementary file 4 — Figure S4 Distribution of Q10 dB values of frequency response areas of FAF and dAC units. [file NYAS-1547-116-s004.tif]

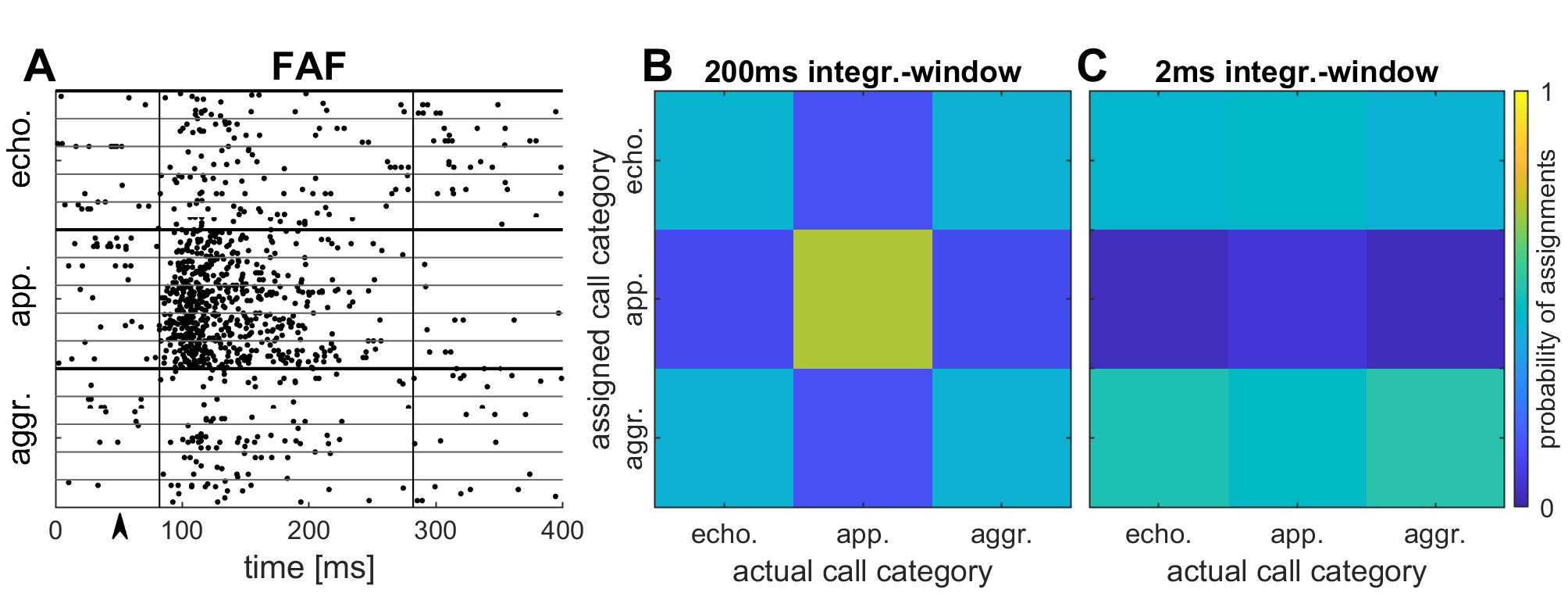

Supplement: Supplementary file 5 — Figure S5 Spike response patterns and confusion matrix for category assignments for the FAF unit shown in Figure 6A. [file NYAS-1547-116-s005.tif]
